# A Numerical Study on Molten Pool Behavior and Ribbon Thickness Under Varying Casting Parameters in Planar Flow Casting

**DOI:** 10.3390/ma19132883

**Published:** 2026-07-06

**Authors:** Lijun Li, Jianliang Sun, Hongxin Ji, Deren Li, Baisong Li, Na Lv, Xianyan Wang, Xiangyu Lv

**Affiliations:** 1 Advanced Technology and Materials Co., Ltd., China Iron and Steel Research Institute Group, Beijing 100081, China; lilijun@atmcn.com (L.L.); libaisong@atmcn.com (B.L.); lvna@atmcn.com (N.L.); wangxianyan@atmcn.com (X.W.); lvxiangyu@atmcn.com (X.L.); 2 National Engineering Research Center for Equipment and Technology of Cold Strip Rolling, Yanshan University, Qinhuangdao 066004, China; 3 School of Electrical Engineering, Beijing Jiaotong University, Beijing 100044, China

**Keywords:** planar flow casting, melt puddle, ribbon thickness, air pockets

## Abstract

Despite the widespread use of planar flow casting (PFC) for amorphous alloy ribbons, previous two-dimensional (2D) numerical studies have primarily focused on isolated flow or thermal behaviors, lacking a systematic quantification of how key casting parameters collectively influence melt puddle geometry and ribbon thickness. To fill this gap, this work establishes a coupled air–melt two-phase 2D Volume of Fluid (VOF) model based on the continuity, momentum, and energy equations. An analysis was conducted on how various parameters affect the melt puddle behavior and ribbon thickness. The results indicate that, as the roller speed (U) increases from 21 m/s to 30 m/s, the detachment length (Ln) decreases by 31%. Over the same interval, the puddle length (L) decreases by 30%. When the ejection speed (V) increases from 1.4 m/s to 2.0 m/s, the ejection temperature ($T_{e}$) increases from 1433 K to 1733 K, and the slit width (W) increases from 0.4 mm to 0.6 mm, Ln rises by roughly 39.7–133%, while L increases by approximately 32.3–112%. To produce thinner amorphous ribbons for loss reduction, high roller speed, low ejection speed, and small nozzle slit are crucial parameters.

## 1. Introduction

**Figure 1:**
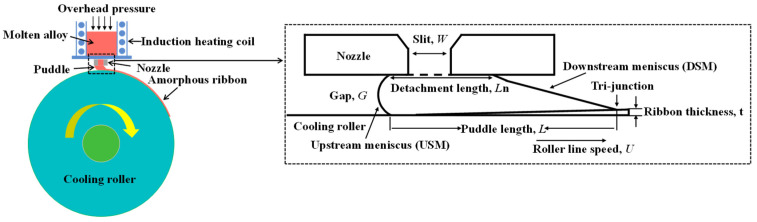
Schematic diagram of the PFC process (not to scale).

As a novel functional material, amorphous alloy ribbons exhibit outstanding physical, mechanical, and magnetic properties and are extensively used in areas such as communication electronics and new energy vehicles [1,2,3]. In the industrial production of amorphous ribbons, planar flow casting (PFC) is a major rapid solidification technique [4]. During the PFC process, the molten alloy is ejected through a nozzle under external pressure onto a rapidly rotating copper cooling roller. Between the nozzle and the roller, a small melt puddle is formed and held by surface tension. When the liquid molten alloy in the puddle comes into contact with the cooling roller, it solidifies into a ribbon at an extremely high cooling rate (~10^6^ K/s), as illustrated in Figure 1.

The fabrication of amorphous ribbons via PFC involves a non-equilibrium solidification process carried out under conditions of high temperature, high speed, and high pressure. This process, which includes complex heat and mass transfer within multiphase flow, along with the coupling of multiple physical fields, makes it difficult to conduct experimental research on the formation process of the melt puddle. Numerical simulation can examine fluid dynamic behavior in melt puddles over a very short timescale and is an efficient tool for research on melt puddles.

Ribbon thickness is proportional to core loss, and thinner amorphous ribbons represent a critical development trend [5]. Therefore, investigating the PFC process parameters to produce thinner ribbons is crucial for improving their industrial application. Previous numerical studies have contributed significantly to understanding melt flow and heat transfer in the PFC process. For instance, Liu et al. [6] performed a 2D simulation of melt flow and heat transfer, while Sowjanya et al. [7] developed a 2D model to study melt puddle formation dynamics. However, most prior 2D simulations have not addressed the coupled air–melt two-phase interfacial dynamics or the risk of air-pocket entrainment, which directly affects ribbon quality and casting stability. Although Ji et al. [8] studied heat and mass transfer using a 3D coupled multiphase model, its high computational cost limits parametric studies. Consequently, there is a lack of numerical guidelines for selecting process parameters that balance thickness reduction with surface defect control.

The motion characteristics of the gas–liquid interface are crucial to the PFC process. Numerical methods for simulating two-phase gas–liquid flows can be categorized into Eulerian and Lagrangian schemes. Among these, the VOF method within the Eulerian framework is a robust technique that can effectively capture complex interfaces [9]. Yan et al. [10] noted that the VOF method can precisely capture gas–liquid interfaces. Li et al. [11] developed an advection–reaction-based interface-sharpening method combined with fully threaded tree adaptive mesh refinement to reduce numerical diffusion in compressible two-phase VOF simulations and improve efficiency. Their results confirm that the VOF model can effectively capture detailed interface dynamics in the PFC casting process.

In this paper, a coupled air–melt two-phase 2D Volume of Fluid (VOF) model is established based on the continuity, momentum, and energy equations. Despite certain simplifications, including constant surface tension, neglected Marangoni effects, and interfacial heat-transfer resistance, along with a lack of experimental validation, this model can still quickly and effectively provide actionable insights for the industrial production of thinner amorphous ribbons by systematically analyzing the individual and combined effects of U, V, $T_{e}$, W, and G on melt puddle behavior, ribbon thickness, and air-pocket formation.

## 2. Numerical Model

### 2.1. Computational Zone and Boundary Conditions

**Figure 2:**
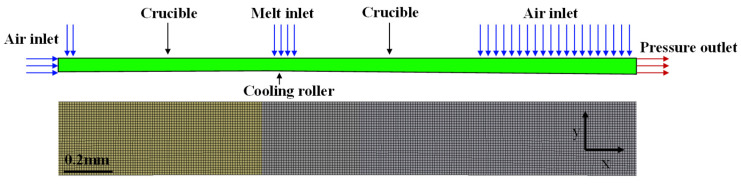
The computational domain and meshing of the model.

As shown in Figure 2, the area between the nozzle and the cooling roller is set as the calculation region, and a global quadrilateral mesh is divided for it with a mesh size of 0.01 mm. Ultimately, 52,173 nodes and 50,642 elements are generated. The boundary conditions for fluid flow and heat transfer at the inlet are set as velocity and constant temperature, respectively. The copper roller surface is maintained at a constant temperature of 300 K to ensure uniform ribbon thickness and amorphous nature [12]. The crucible wall is given a non-slipping velocity boundary condition and an adiabatic heat transfer condition. The other boundaries that constitute the computational domain are the atmosphere. The atmosphere is set as the pressure inlet and outlet boundary, with a relative pressure of 0. The initial temperature settings for other materials are 300 K, except for the amorphous alloy. The roller diameter is 600 mm, and the model domain spans x from −5.7 mm to 9.3 mm. This range is sufficient to ensure proper development of the melt puddle.

### 2.2. Fundamental Assumptions

(1) Since the ribbon’s width and length are far greater than its thickness, and flow and heat transfer are uniform across the width direction, a two-dimensional model effectively captures the key phenomena while reducing computational cost.

(2) In the narrow nozzle–roller gap, no significant turbulence occurs, and laminar flow accurately represents the actual flow state of both the melt and the air.

(3) Most material properties vary only slightly over the working temperature range. Assuming them constant simplifies calculations, while only the temperature-dependent melt viscosity is retained for accuracy.

(4) The ultra-high cooling rate leads to extremely rapid solidification. Phase-change latent heat exerts a negligible effect on temperature and flow fields.

### 2.3. Mathematical Model

The following equations were applied in the simulation [13]:

Continuity equation:

(1) $$\frac{\partial \rho }{\partial t}+\frac{\partial (\rho u_{i})}{\partial x_{i}}=0$$

The volume fraction F follows the equations presented below.

(2) $$\frac{\partial F}{\partial t}+u_{i}\frac{\partial F}{\partial x_{i}}=0$$

(3) $$F=\left\{\begin{array}{l} 0,The\;entire\;calculation\;zone\;is\;filled\;with\;air\\ 0<F<1,air+melt\\ 1.0,The\;entire\;calculation\;zone\;is\;filled\;with\;melt \end{array}\right.$$

In the transport equations, the thermophysical properties of air and melt are governed by the value of F within each cell, which can be determined using the following equations:

(4) $$\begin{array}{l} \rho =\rho_{a}F+\rho_{m}\left(1-F\right)\\ \mu =\mu_{a}F+\mu_{m}\left(1-F\right)\\ C_{p}=C_{p,a}F+C_{p,m}\left(1-F\right)\\ K=K_{a}F=K_{m}\left(1-F\right) \end{array}$$

The subscripts ‘a’ and ‘m’ denote air and amorphous material, respectively. K represents the thermal conductivity (W·m^−1^·K^−1^).

Momentum equation:

(5) $$\rho (\frac{\partial (u_{i})}{\partial t}+\frac{\partial (u_{i}u_{j})}{\partial x_{j}})=\frac{\partial }{\partial x_{j}}\left[\mu \left[\frac{\partial u_{i}}{\partial x_{j}}+\frac{\partial u_{j}}{\partial x_{i}}\right]\right]-\frac{\partial P}{\partial x_{i}}+\rho g+f$$

(6) $$f=\sigma \frac{\rho \kappa \nabla F}{\frac{1}{2}\left(\rho_{a}+\rho_{m}\right)}$$

(7) $$\kappa =-\nabla \cdot \frac{\nabla F}{\left|\nabla F\right|}$$

where ρ is the density (kg/m^3^), t represents time (s), $u_{i}$ and $u_{j}$ denote the velocity components (m/s), $x_{i}$ and $x_{j}$ signify the directional components, μ is the viscosity (Pa·s), P represents the pressure (Pa), g is the gravitational acceleration (m/s^2^), f refers to the surface tension source phase, and κ is curvature of the interface.

Energy equations:

(8) $$\rho (\frac{\partial T}{\partial t}+\frac{\partial \left(C_{p}u_{i}T\right)}{\partial x_{i}})=\frac{\partial }{\partial x_{i}}\left(K\frac{\partial T}{\partial x_{i}}\right)$$

(9) $$\frac{\partial }{\partial_{t}}\left(\rho_{cr}C_{p,cr}T\right)+\frac{\partial }{\partial x_{i}}\left(\rho_{cr}C_{p,cr}{\left(\omega R\right)}_{i}T\right)=\frac{\partial }{\partial x_{i}}\left(K_{cr}\frac{\partial T}{\partial x_{i}}\right)$$

where $C_{p}$ denotes the constant pressure specific heat (J·kg^−1^·K^−1^), T is temperature (K), cr represents the roller, ω indicates the rotation speed of the roller (rad/s), and R signifies the radius of the roller (m).

The viscosity expression of the amorphous alloy is expressed as follows [14]:

(10) $$\mu =0.10\times \exp\left(-3.6528+734.1/\left(T-674\right)\right)$$

The solid–liquid interface is defined by the melt isotherm at 873 K. The 873 K isotherm marks the melt solidification boundary under the ultra-fast cooling of PFC. The vertical distance from this isotherm at the tri-junction (J*) to the roller surface directly represents the final ribbon thickness. In this work, the J* is defined as the intersection of the downstream meniscus with this isotherm, and the ribbon thickness is taken as the vertical distance from J* to the roller surface.

### 2.4. Calculation Conditions and Thermophysical Parameters

The different simulation conditions of this article are shown in Table 1. The thermal physical property parameters of amorphous alloy are presented in Table 2. The physical properties of air and copper roller are taken from the system material library.

### 2.5. Numerical Simulation Method

ANSYS Fluent 2021 R1 was used to conduct the simulation analysis. The time step was set at 1 × 10^−6^ s, and the calculation time was 30 milliseconds. A transient pressure-based coupling solver was established using PISO pressure–velocity coupling. The momentum and energy equations were solved using the first-order upwind scheme. Although this scheme may introduce slight numerical diffusion, it features good numerical stability and robustness for the complex two-phase flow and solidification problems in planar flow casting, which ensures reliable calculation results. A geometric reconstruction scheme tracked the air–melt interface. Mesh sensitivity analysis is conducted, and the results are given in Appendix A.

## 3. Result and Discussion

### 3.1. Simulation Analysis of the PFC Process

#### 3.1.1. The Process of Melt Puddle Formation

**Figure 3:**
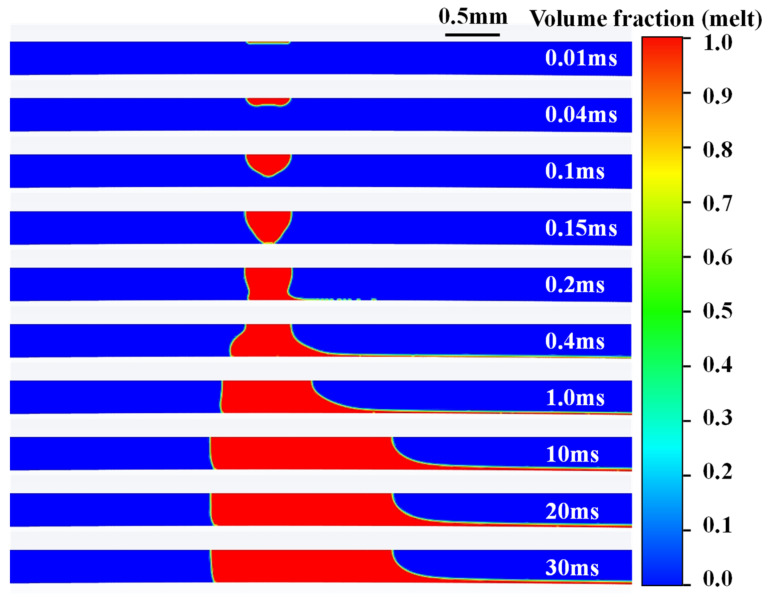
The shapes of the melt puddle at different times.

Figure 3 shows the shapes of the melt puddle at different times under simulation case 1 (the red zone represents the molten metal, and the blue zone indicates the air). The transition from initial impact to a quasi-steady melt puddle consists of three physical stages. First, after the melt is ejected from the nozzle slit, surface tension dominates its shape, creating a central depression at about 0.04 ms. As inertia overcomes surface tension (around 0.1 ms), the melt front develops into a V-shaped jet. Second (0.1–0.2 ms), the tip of the V-shaped jet contacts the cooling roller surface. The roller’s high-speed rotation exerts a strong shear drag, causing rapid downstream spreading, while continuous melt supply forms an upward-curved crescent in the upstream region. Third (0.2–10 ms), the upstream meniscus (USM) reaches a dynamic balance between surface tension and static pressure, stabilizing into a crescent shape. Meanwhile, the downstream meniscus (DSM) is governed by roller drag and melt outflow, evolving into a stable slope-like shape.

#### 3.1.2. Temperature Field Distribution at Different Times

**Figure 4:**
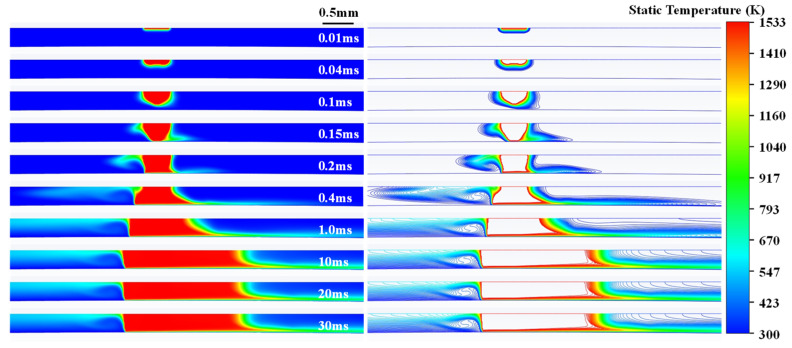
Distribution of temperature field at different times.

Figure 4 presents the temperature field distribution at various times in simulation case 1. At t = 0.04 ms, only the air adjacent to the melt was heated, approaching the ejection temperature. At t = 0.1 ms, the melt temperature remained unchanged, while convective heat transfer raised the temperature of the nearby air. At t = 0.15 ms, airflow increased the temperature on the right side of the melt puddle near the cooling roller. Between 0.2 ms and 1 ms, heat transfer progressively expanded the heat-affected zone, raising the temperature of the air flowing over the melt. At t = 10 ms, the temperature distribution stabilized, with layered isothermal lines forming near the cooling roller.

### 3.2. The Influence of Parameters on the PFC Process

#### 3.2.1. The Influence of Each Parameter on the Characteristics of the Melt Puddle

**Figure 5:**
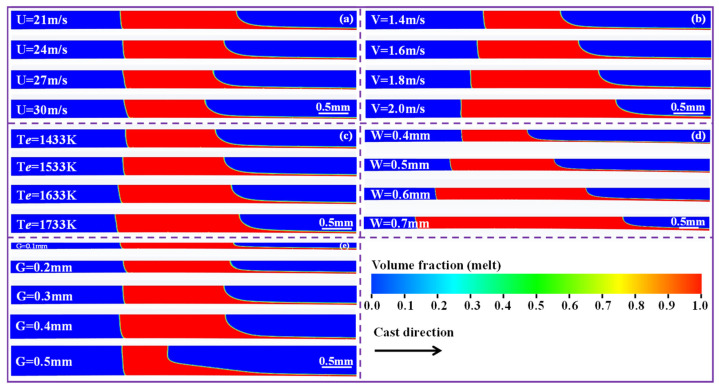
Melt puddle geometries under various simulation conditions. (**a**) Roller speeds; (**b**) Ejection speed; (**c**) Ejection temperature; (**d**) Slit width; (**e**) Gap.

Figure 5 shows the variation in melt puddle geometry under different casting parameters. With all the other parameters held constant, increasing the roller speed reduces the melt puddle size. Both the USM and DSM move toward the center of the nozzle slit, with the USM exhibiting a smaller offset than the DSM. At higher roller speeds, the molten metal is extracted from the melt puddle at a greater velocity, causing the DSM to shift further toward the center of the nozzle slit (Figure 5a). Meanwhile, because the melt injection flow rate remains constant, the USM moves only slightly toward the slit center. Additionally, the melt puddle size increases with rising V (Figure 5b). This is due to the increased V, which boosts the melt flow rate and allows more melt to diffuse into the domain bounded by the nozzle and the roller surfaces. The melt puddle size increases slightly with higher $T_{e}$ due to reduced melt viscosity and improved fluidity (Figure 5c). A wider nozzle slit leads to a notable increase in puddle size (Figure 5d). As the gap (G) decreases, the puddle size also increases (Figure 5e). The smaller G reduces the melt accommodation volume, forcing the melt to expand sideways and lengthen the puddle. When G > W (G = 0.5 mm), DSM is almost perpendicular to the nozzle wall, resulting in the melt flowing almost vertically [15,16].

**Figure 6:**
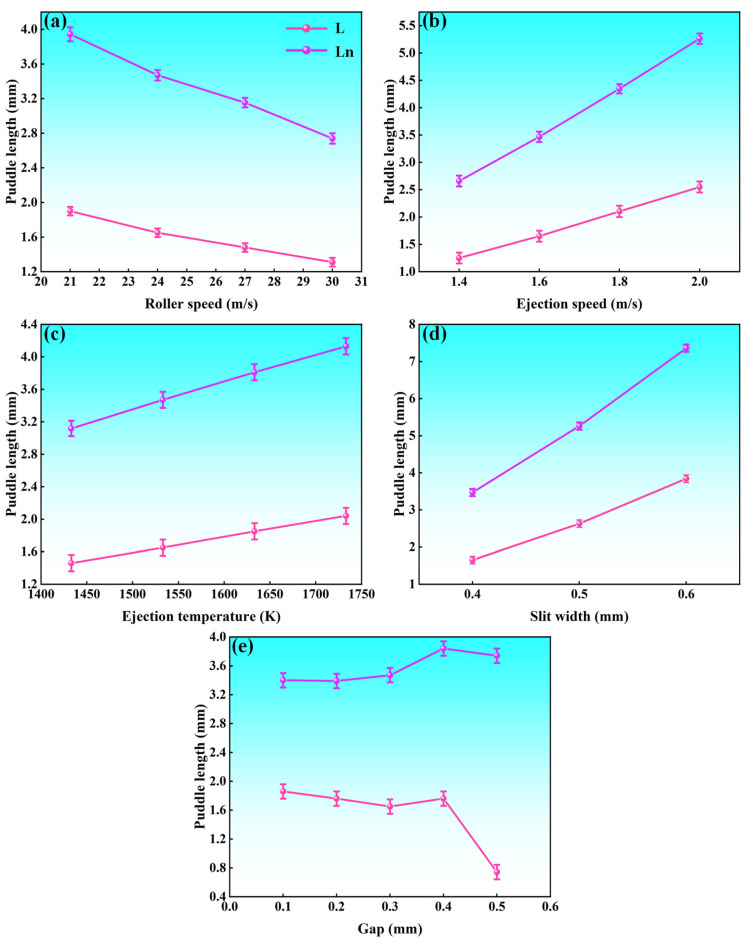
The size of the melt puddle under different simulation conditions. (**a**) Roller speeds; (**b**) Ejection speed; (**c**) Ejection temperature; (**d**) Slit width; (**e**) Gap.

Increasing U from 21 m/s to 30 m/s reduced Ln from 1.9 mm to 1.31 mm and L from 3.93 mm to 2.74 mm (Figure 6a). Conversely, raising V from 1.4 m/s to 2.0 m/s increased Ln from 1.25 mm to 2.55 mm and L from 2.66 mm to 5.26 mm (Figure 6b). Raising $T_{e}$ from 1433 K to 1733 K increased Ln from 1.46 mm to 2.04 mm and L from 3.12 mm to 4.13 mm (Figure 6c). Widening W from 0.4 mm to 0.6 mm raised Ln from 1.65 mm to 3.84 mm and L from 3.47 mm to 7.36 mm (Figure 6d). At a slit width of 0.7 mm, the molten alloy volume and flow rate become so large that no 873 K isothermal line appears. Consequently, the melt cannot cool down to 873 K within the calculated region, indicating incomplete solidification and a potential risk of casting defects or production failure. As G increased from 0.1 mm to 0.5 mm, Ln first dropped from 1.86 mm to 1.65 mm, then rose to 1.76 mm, and finally fell to 0.745 mm; L first rose from 3.4 mm to 3.84 mm and then fell to 3.74 mm (Figure 6e). Within a specific range, increasing U reduces Ln by 31%. Within the same range, the L value decreases by 30%. As V and W increase, Ln rises by approximately 39.7–133% within the specific range, while L increases by about 32.3–112%.

#### 3.2.2. The Effect of Various Parameters on Ribbon Thickness

**Figure 7:**
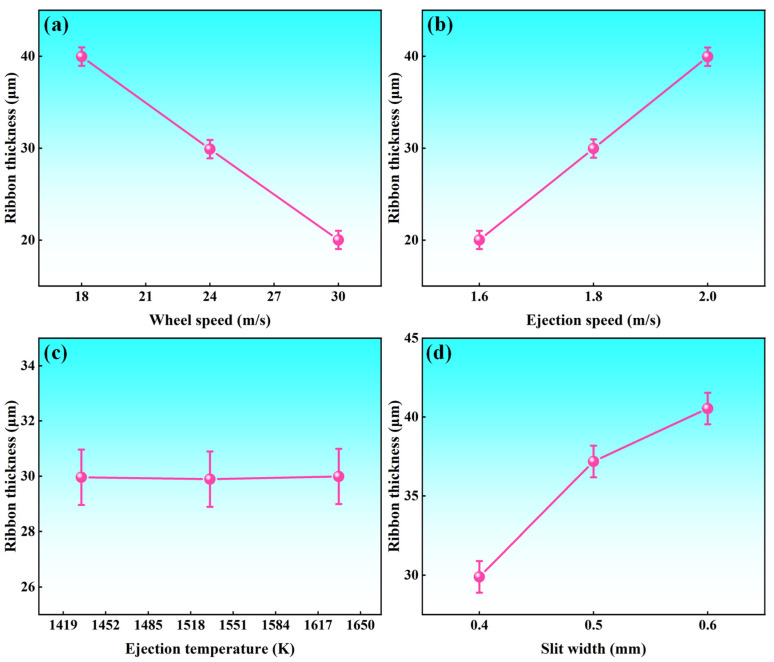
Ribbon thickness under different simulation conditions. (**a**) Roller speeds; (**b**) Ejection speed; (**c**) Ejection temperature; (**d**) Slit width.

As shown in Figure 7a, ribbon thickness decreases with an increase in roller speed. Neither the qualitative trends nor the quantitative values obtained in this study agree with those reported previously [8,17]. Increasing U from 18 m/s to 30 m/s reduces the ribbon thickness from 39.96 μm to 20.02 μm, a 49.9% decrease. This is attributed to the higher roller speed, which accelerates cooling and shortens solidification time, making it essential for producing thinner amorphous ribbons. In contrast, as V rises from 1.6 m/s to 2.0 m/s, the thickness increases from 20.02 μm to 39.95 μm (a 98.7% increase), as shown in Figure 7b. As shown in Figure 7c, the $T_{e}$ has little effect on ribbon thickness. Increasing W from 0.4 mm to 0.6 mm raises the thickness from 29.89 μm to 40.54 μm, a 35.6% increase (Figure 7d). In conclusion, high roller speed, low ejection speed, and a narrow nozzle slit are key for producing thinner amorphous ribbons.

#### 3.2.3. The Formation of Air Pockets in the Melt Puddle

**Figure 8:**
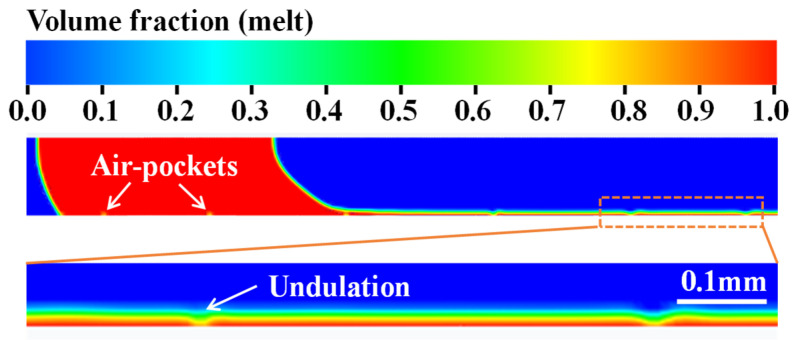
Air pockets in the melt puddle and surface undulations.

As shown in Figure 8, air pockets will be entrained at the contact position between the USM and the cooling roller surface when the roller speed is 54 m/s. This affects the stability of the melt puddle, causing periodic undulations on the ribbon surface and impacting its forming quality, which can lead to material waste. In severe cases, this can result in casting failure [18]. As can be seen, producing thinner ribbons is not as simple as increasing the roller speed indefinitely. Instead, the required ribbon thickness must be balanced with ribbon quality by considering various casting ribbon parameters.

**Figure 9:**
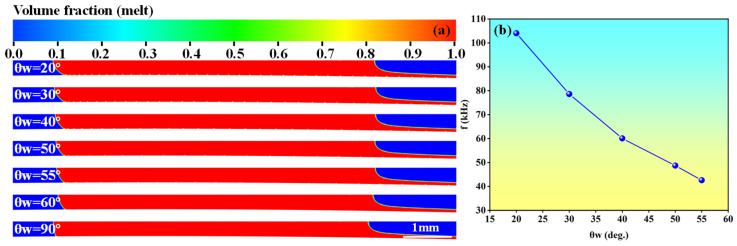
The frequency of air pockets in the molten pool under different θw. (**a**) Contour maps of melt volume fraction for θw ranging from 20° to 90°; (**b**) Relationship between air-pocket frequency *f* and contact angle θw.

The appearance of air pockets in the melt puddle can significantly affect the magnetic properties of the ribbons [19]. The wetting condition of the roller surface is an important factor affecting the frequency of air pockets. Using the static contact angle in the wall adhesion model of the VOF method, the effect of roller surface wettability on bubble behavior in the melt pool was examined. The wetting state can be controlled by changing the contact angle of the melt–roller surface (θw). The air-pocket frequency was determined from periodic observations. Specifically, the average distance between two adjacent air pockets was measured and divided by the roller speed to obtain the time interval; the reciprocal of this interval then gave the air-pocket frequency. At θw ≤ 90°, when θw increased from 20° to 55° (Figure 9a), the frequency of air-pocket entrainment in the molten pool decreased from 104 kHz to 42.6 kHz (Figure 9b). When θw = 60°, air pockets no longer appear in the melt puddle.

## 4. Conclusions

In this paper, a 2D multiphase flow coupling model for the preparation of amorphous ribbons by PFC was established. The influences of different parameters on the characteristics of a melt puddle and ribbon thickness were analyzed, and the following conclusions were obtained:

(1) From the initial ejection of the melt to the establishment of a quasi-steady state, the process successively undergoes three stages. These include contraction dominated by surface tension, formation of a V-shaped jet under inertial force, and spreading driven by the shear dragging of the cooling roller. Ultimately, the upstream meniscus stabilizes into a crescent shape, while the downstream meniscus assumes a sloping configuration, thereby providing the geometric foundation for continuous ribbon formation.

(2) Higher roller speed compresses the melt puddle. Larger ejection speed and slit width expand it upstream and downstream. A small gap forces lateral expansion; a large gap causes vertical fall and shortens the puddle. Higher ejection temperature reduces viscosity and improves fluidity, but its effect on puddle size is moderate.

(3) High roller speed, low ejection speed, and a narrow slit are key for thinner ribbons. Roller speed and ejection speed matter most, while ejection temperature has little effect. However, excessively high roller speed entrains air and forms cavities, damaging quality. Too wide a slit may cause incomplete solidification and casting failure. Thus, thinning must stay within stable forming limits.

(4) At θw ≤ 90°, when increased from 20°to 55°, the frequency of air-pocket entrainment in the molten pool decreased from 104 kHz to 42.6 kHz. When θw = 60°, air pockets no longer appear in the melt puddle. The quantitative link between air-pocket entrainment frequency and the ribbon’s mechanical and magnetic performance remains unconfirmed. Follow-up physical casting trials are needed to verify whether tuning bubble formation can reliably tailor the final functional properties of as-cast ribbons.

## Figures and Tables

**Table 1:** Simulation conditions.

Simulation Case No.	Ejection Speed (V) (m/s)	Ejection Temperature ($T_{e}$) (K)	Roller Speed (U) (m/s)	Slit Width (W) (mm)	Gap (G) (mm)
1	1.6	1533	24	0.4	0.3
2	1.4	1533	24	0.4	0.3
3	1.8	1533	24	0.4	0.3
4	2.0	1533	24	0.4	0.3
5	1.6	1433	24	0.4	0.3
6	1.6	1633	24	0.4	0.3
7	1.6	1733	24	0.4	0.3
8	1.6	1533	21	0.4	0.3
9	1.6	1533	27	0.4	0.3
10	1.6	1533	30	0.4	0.3
11	1.6	1533	24	0.5	0.3
12	1.6	1533	24	0.6	0.3
13	1.6	1533	24	0.7	0.3
14	1.6	1533	24	0.4	0.1
15	1.6	1533	24	0.4	0.2
16	1.6	1533	24	0.4	0.4
17	1.6	1533	24	0.4	0.5

**Table 2:** Thermal physical properties of amorphous alloy, air and copper roller.

Materials	ρ (kg/m^3^)	$C_{p}$ (J·kg^−1^·K^−1^)	*K* (W·m^−1^·K^−1^)	μ (Pa·s)
Amorphous alloy	7180	544	8.99	Equation (10)
Air	1.225	1006.43	0.0242	1.7894 × 10^−5^
Cooling roller	8978	385	400	—

## Data Availability

The original contributions presented in this study are included in the article. Further inquiries can be directed to the corresponding authors.

## Appendix A

ANSYS 2021 R1 Meshing was employed to generate a high-quality hexahedral mesh for the computational domain. A mesh independence analysis was performed under the following conditions: melt ejection speed V = 1.6 m/s, ejection temperature *T_e_* = 1623 K, wheel speed U = 24 m/s, slit width W = 0.4 mm, and nozzle–wheel gap G = 0.3 mm. The resulting ribbon thicknesses for different mesh sizes are listed in Table A1. The simulation used a time step of 1 × 10^−6^ s and a total duration of 20 ms. As shown in Table A1, the ribbon thickness values obtained with Mesh 2 and Mesh 3 are nearly identical, with a negligible difference. Therefore, Mesh 2, which contains 50,642 elements, was selected to reduce computational time.

**Table A1:** Ribbon thickness variations under different meshes.

Mesh	No. of Elements	Ribbon Thickness (μm)
1	12,597	40.35
2	50,642	30.12
3	202,217	29.98
